# DYADIC PROFILES OF COUPLES’ SELF-PERCEPTIONS OF AGING: IMPLICATIONS FOR MENTAL HEALTH

**DOI:** 10.1093/geroni/igad104.1209

**Published:** 2023-12-21

**Authors:** Meng Huo, Kyungmin Kim

**Affiliations:** University of California, Davis, Davis, California, United States; Seoul National University, Seoul, Republic of Korea

## Abstract

The way older adults perceive their own aging processes influences their mental health, but we know little about how this occurs in a dyadic context, where spouses’ perceptions and health are often intertwined. The current study aims to identify dyadic profiles of self-perceptions of aging (SPA) in couples and examine how certain dyadic profiles are associated with each partner’s mental health over time. A pooled sample of 3,850 heterosexual couples aged 50+ in the Health and Retirement Study (2012/2014–2016/2018) rated positive and negative SPA and provided data on demographic characteristics, couple relationships, and health. Latent profile analysis revealed four profiles of couples’ SPA: similarly positive (32%), similarly negative (10%), wife positive–husband negative (38%), and wife negative–husband positive (20%). Physical health and marital quality differentiated couples and predicted profile membership. Couples with similarly positive SPA reported fewer depressive symptoms over time than did couples in other profiles; couples with similarly negative SPA fared worse mental health. We observed a gender difference when comparing the two opposite profiles, such that wives in the wife positive–husband negative profile reported increasingly fewer depressive symptoms than wives in the wife negative–husband positive profile. Husbands in those two profiles did not differ in mental health. This study adds to the emerging literature that advocates for an interpersonal approach to SPA. Findings revealed risk and resilience in couples as they experience and perceive aging and inform dyadic interventions that aim to protect older adults’ mental health in the long run.

